# Real-Time Data Acquisition System for Array MIMU Based on FPGA+ARM

**DOI:** 10.3390/mi17020239

**Published:** 2026-02-12

**Authors:** Xiaoyang Qin, Huan Wang, Zhihua Dai, Yonghua Wang, Junqing Zhang, Tao Guo, Huiliang Cao

**Affiliations:** 1 State Key Laboratory of Extreme Environment Optoelectronic Dynamic Measurement Technology and Instrument, North University of China, Taiyuan 030051, China; 2 School of Instrument and Electronics, North University of China, Taiyuan 030051, China; 3 Quanzhou Yunjian Measurement Control and Perception Technology Innovation Research Institute, Quanzhou 362000, China; 4 School of Civil Engineering, Tangshan University, Tangshan 063000, China; 5 Tangshan Zhongyu Technology Development Co., Ltd., Tangshan 063000, China; 6 School of Integrated Circuits and Electronics, Beijing Institute of Technology, Beijing 100081, China; 7 Bit Chongqing Institute of Microelectronics and Microsystems, Chongqing 400000, China

**Keywords:** MIMU, array-based, real-time, FPGA, ARM, SPI protocol

## Abstract

To address the issue of low accuracy and stability in the gyroscope components of the micro-inertial-measurement-unit (MIMU) core units, which limits their application in high-precision scenarios, this paper designs a real-time data acquisition system for array MIMU based on FPGA and ARM. This system establishes a complete data chain encompassing raw data acquisition, real-time processing, multi-source information fusion, data storage, and communication with a host computer. It has been successfully applied to a 100-m pipeline position coordinate measurement scenario. The paper begins by discussing the overall system design, including both hardware circuit and software code development. Attitude update algorithms and measurement accuracy evaluation metrics are also introduced. System functionality is validated through static tests and practical pipeline measurements. Experimental results demonstrate that the system improves the accuracy of a single micro-electro-mechanical system (MEMS) gyroscope by a factor of 7.4 to 7.7. It also enables precise calculation of the pipeline position coordinates over the 100 m distance, achieving a horizontal positioning error of less than 0.0774 m and an elevation positioning error of less than 0.0351 m. These results fully confirm the significant effectiveness of the array design in mitigating gyroscope random errors, providing a reliable technical solution for pipeline measurement.

## 1. Introduction

Within inertial navigation systems, the micro-inertial-measurement-unit (MIMU) serves as the core component across numerous navigation systems. It incorporates a gyroscope and accelerometer on each of three orthogonal axis, typically employed to measure angular velocity and acceleration during navigation [1,2,3]. Compared to conventional mechanical sensors, the MIMU has garnered significant attention in recent years due to its advantages of low cost, compact size, and ease of integration, leading to substantial market demand. Consequently, it has become an essential component in diverse navigation systems, attitude control devices, unmanned aerial vehicles, robotic navigation, and satellite systems [4,5,6]. Despite their extensive applications, MIMUs suffer from relatively low precision and stability due to the frequent influence of environmental, electronic, and manufacturing noise on micro-electro-mechanical system (MEMS) gyroscopes. This limitation restricts their use in scenarios demanding high accuracy.

To enhance the accuracy of gyroscope components within MIMUs, optimisation of manufacturing processes and digital signal processing techniques are typically employed. However, improving the precision of micro-mechanical structures through process refinement significantly increases sensor production costs, and substantial accuracy gains via structural modifications prove challenging. Therefore, forming an array circuit by calibrating and compensating MEMS gyroscopes, followed by data fusion to achieve higher measurement accuracy, represents a cost-effective approach. This technique, which constructs high-precision MEMS gyroscopes through array formation using MEMS gyroscopes and data fusion technology while simultaneously reducing drift noise, is termed ‘virtual gyroscope’ technology [7,8,9,10].

Gyroscope array technology was first proposed by Bayard et al. at the US Jet Propulsion Laboratory (JPL) and termed the ‘virtual gyroscope’ [11]. In 2003, Bayard et al. pioneered a virtual gyroscope technique that fused data from four identical MEMS gyroscopes. Experimental validation demonstrated the method’s feasibility, achieving approximately double the accuracy. They further concluded that when N identical yet uncorrelated gyroscopes collect data under identical conditions, the drift resulting from fusing their data reduces to $\sqrt{N}$ times the original value. Kamata et al. proposed a small FPGA-based array board comprising 32 MEMS gyroscopes, reducing bias stability from 2.2 °/h to 0.5 °/h [12]. Wang et al. employed a gyro array of 72 MPU-6000 sensors, achieving approximately 50 reduction in angular error after 20 runs over one hour—effectively halving the fusion error Kamata2020. Dominguez et al. designed and implemented a differential IMU array using 16 ASM330 MEMS sensors, yielding a high-precision virtual gyroscope output [13]. Domestically, Yi et al. designed and implemented a differential IMU array comprising 16 ASM330 MEMS sensors, achieving a high-precision virtual gyroscope output [14]. However, the aforementioned research circuits predominantly employ ARM processors as microcontrollers, sequentially reading sensor data. This sequential approach introduces temporal differences, making it challenging to ensure synchronous data measurement timing. Presently, controllers in data acquisition systems predominantly employ ARM processors, which execute tasks sequentially by processing commands one by one. When utilised as the primary controller for reading gyroscope array data, the inherent programme execution delay makes it challenging to obtain data from the gyroscope array at precisely the same moment.

Therefore, in order to enhance the performance of MEMS gyroscopes and enable simultaneous reading of multiple sensors, this paper designs and implements a real-time signal acquisition system for MIMU arrays based on FPGA+ARM architecture, applied to pipeline measurement scenarios. The system employs FPGA and ARM working in tandem, with the FPGA synchronously acquiring and fusing 60-channel MIMU data via SPI bus. Raw acquisition data is transmitted to the ARM processor through a serial port, where the ARM handles data parsing, multi-source information fusion, data storage, and external communication interactions.

The remaining structure of this paper is organised as follows: Section 2 establishes the overall design scheme for the array MIMU real-time data acquisition system, covering core elements such as hardware circuit design and software code development. Section 3 elaborates on the quaternion-based attitude update method and position update method, introducing relevant evaluation metrics. Section 4 validates and analyses the proposed data acquisition system through static experiments and practical pipeline scenario testing. Section 5 summarises the entire paper and outlines future research directions.

## 2. Overall Design of the Acquisition System

This chapter shall present the design rationale for the two core components of the array MIMU system, encompassing both hardware circuitry and software code design. Adopting a top-level architectural concept of ‘distributed acquisition, centralised processing, and unified transmission’, the objective is to establish a high-precision hardware measurement system capable of synchronously acquiring multiple MIMU data streams.

### 2.1. Critical Processor Selection and Justification

Currently, mainstream MIMUs predominantly support the SPI communication protocol, necessitating the selection of a main controller capable of driving multiple SPI interfaces in parallel. Traditional MCUs typically employ analogue switches to toggle chip select signals for time-division multiplexing of SPI interfaces. This approach is prone to data blocking and transmission delays, severely compromising system synchronisation accuracy and real-time performance, and proving inadequate for synchronous reading of multiple MIMUs. Conversely, FPGAs leverage programmable logic resources to instantiate multiple independent SPI controller cores via software programming, enabling simultaneous data acquisition from multiple MIMUs.

### 2.2. System Hardware Circuit Design

**Figure 1:**
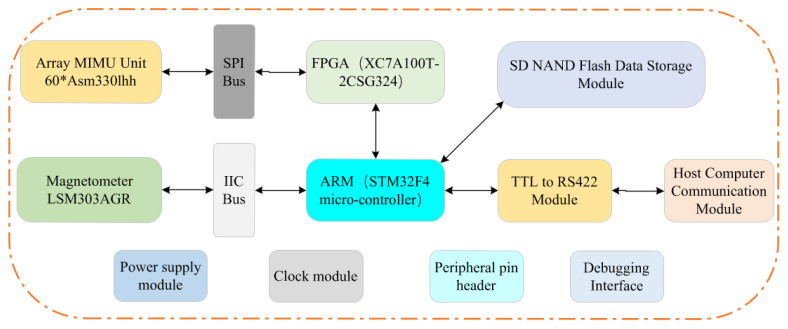
Hardware design of an array MIMU data acquisition system based on FPGA+ARM.

The MIMU array system design comprises multiple modules, with its structural block diagram illustrated in Figure 1. This figure presents an array MIMU data acquisition system based on FPGA+ARM, encompassing the MIMU data acquisition module (based on the SPI bus), magnetometer data acquisition module (based on the I^2^C bus), STM32F4 and FPGA main control modules, SD NAND Flash storage module, and RS422 communication module. Regarding hardware layout, considering signal integrity and PCB size constraints, the system employs a split-board design: the 60-channel MIMU is integrated onto one PCB. The FPGA is deployed independently on a separate PCB, responsible for synchronous acquisition and real-time averaging processing of the 60-channel MIMU data. While the STM32F4 is configured on another independent PCB, undertaking core functions such as data updating, storage, and transmission.

The MIMU array comprises sixty ASM330 sensors, each capable of capturing triaxial angular velocity and triaxial acceleration data. All data is synchronously transmitted back to the FPGA for processing via the SPI communication interface’s master-to-slave input (MOSI) line. Operating within a 3.3V voltage range, the system employs a TLV75533PDBVR voltage regulator to convert 5 V power to 3.3 V. Capacitors are utilised to filter out alternating frequency components within the DC voltage.

For the designed array system, simultaneous reading of 60 sensors requires at least 60 GPIO ports for the MOSI lines. Regarding the master-to-slave output (MISO) line, clock (CLK) line, and chip select (CS) line, since synchronous reading of 60 sensors is required, a single MISO, CLK, and CS line can synchronously control all 60 sensors. Considering that the MISO, CLK, and CS lines must simultaneously drive 60 sensors, the SN74AUP3G34 buffer is employed here to enhance the driving capability of these lines and ensure signal integrity.

**Figure 2:**
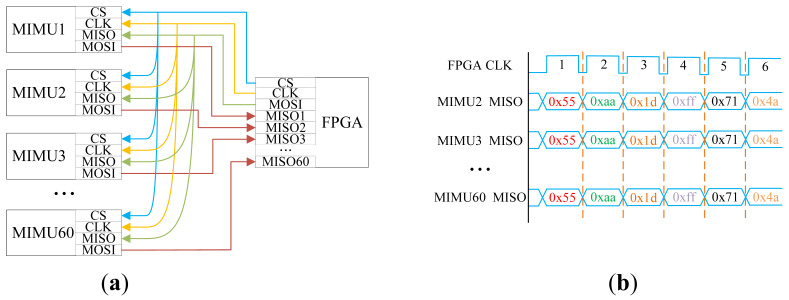
SPI bus topology of the array MIMU and SPI timing diagram for FPGA: (**a**) SPI bus topology of the array MIMU; (**b**) SPI timing diagram for FPGA.

The SPI bus topology employed in this design is illustrated in Figure 2a. This configuration connects all MIMU MISO, CLK, and CS lines in parallel to corresponding pins on the FPGA. The FPGA’s MOSI line is broadcast-connected to all MIMU MISO lines, enabling unified transmission of command data. Concurrently, each MIMU’s MOSI line connects independently to a dedicated FPGA pin, thereby forming 60 distinct parallel data return channels. This topology ingeniously integrates broadcast write and parallel read mechanisms: during the write phase, the FPGA synchronously transmits configuration commands or read instructions to all MIMUs via the shared MOSI line. During the data read phase, the FPGA can simultaneously capture data from all 60 MIMUs, timing diagram as shown in Figure 2b. Within the same clock cycle, it performs data acquisition on multiple sensors concurrently, ensuring the system’s real-time capability.

Compared to traditional daisy-chain or star topologies, this design significantly optimises the real-time parallel data acquisition capability of multi-sensor systems while fully utilising FPGA pin resources.

**Figure 3:**
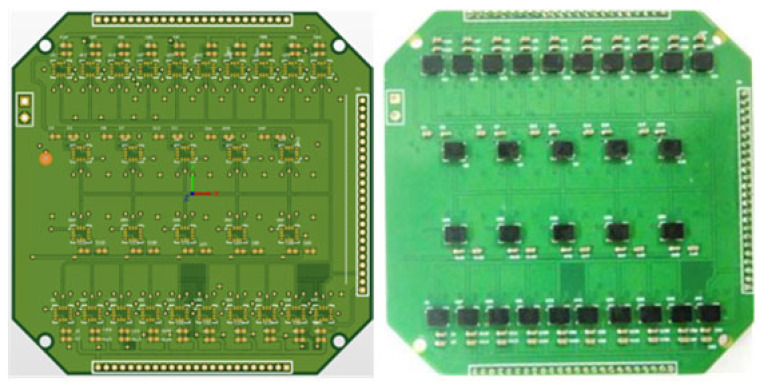
Schematic diagram of front layout and physical image of the array MIMU PCB.

To further enhance the performance of the fused virtual MIMU while balancing system routing complexity and manufacturing costs, the MIMU array design integrates 60 sensors in a spatially symmetric configuration, as illustrated in Figure 3. Thirty sensors are arranged on each side of the PCB, with their Z-sensitive axis oriented in mirror symmetry. Within the same board surface, the sensors are further divided into upper and lower groups of 15 each, with their X and Y sensitive axes arranged in vertical symmetry. This configuration not only effectively mitigates the impact of interference such as temperature drift, board vibration, and circuit noise on MIMU measurement results, thereby improving test accuracy, but also provides ample measurement information to enhance system reliability.

### 2.3. Software Code Design

**Figure 4:**
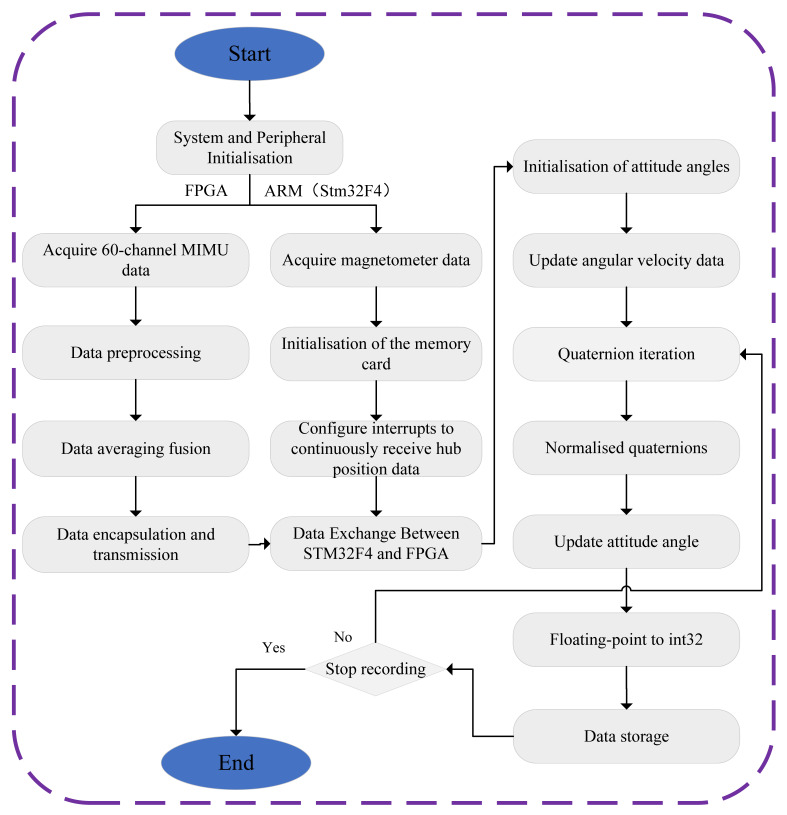
MIMU array system software code flowchart.

The software development for the entire MIMU array system employs a dual-platform architecture based on Vivado and STM32CubeIDE. Specifically, the FPGA logic and control programmes are developed within the Vivado environment to achieve synchronised acquisition and data processing across multiple MIMUs. Meanwhile, the embedded programmes for the ARM processor rely on the STM32CubeIDE environment, responsible for tasks including data reception, attitude angle initialisation, quaternion iteration calculations, updating attitude angles, and data storage. The two components exchange data via the UART serial communication protocol, forming a complete heterogeneous processing system. The software programme execution flow is illustrated in Figure 4.

The FPGA algorithm centres on hardware parallelism to achieve high-synchronisation data acquisition and primary fusion for 60 MIMUs: upon system power-up, the FPGA first configures the global clock and initialises the custom SPI controller IP core. This IP core generates 60 independent SPI control signals to drive each MIMU sensor in parallel. Subsequently, this IP core transmits synchronised acquisition commands to all MIMUs in parallel and receives raw data. The hardware-level synchronisation mechanism eliminates the time lag inherent in traditional sequential acquisition via MCU, ensuring consistent data timestamps. The FPGA then validates the raw data and performs real-time averaging fusion of all 60 measurement channels at the hardware level, generating a high signal-to-noise ratio data stream that substantially reduces the computational load on the backend. Finally, the pre-processed fused data is encapsulated into fixed-length frames and transmitted via UART at 921,600 bps to the STM32F4 microcontroller (Manufacturer: STMicroelectronics; Corporate Location: Geneva, Switzerland).

The ARM receives the fused data from the FPGA and, combined with magnetometer information, performs attitude calculation and storage: the UART interrupt service routine parses the data frame, extracts the fused angular velocity and acceleration data, then fuses the triaxial magnetometer data to complete attitude initialisation (calculating the initial attitude quaternion using static accelerometer and magnetometer data). Subsequently, the quaternion is updated in real-time based on the fused angular velocity and undergoes normalisation processing. This converts it into roll, pitch, and yaw angles. The floating-point attitude angles are then converted to int32 format to accommodate the storage format. Finally, the attitude angles, carrier travel distance, and raw inertial data are written to the storage chip, forming a complete closed-loop process of ‘data acquisition-processing-storage’.

## 3. Theoretical Research

### 3.1. Quaternion-Based Method for Attitude and Position Update

Defining the carrier coordinate system as b and the navigation coordinate system as n, the coordinate transformation matrix $C_{b}^{n}$ from b to n is termed the attitude transformation matrix. As the MIMU is rigidly attached to the carrier, all measured attitude data are based on the carrier coordinate system. Therefore, during attitude estimation, the attitude update matrix $C_{b}^{n}$ is required to convert data from the carrier coordinate system to the navigation coordinate system. In fields such as attitude estimation and navigation, quaternions are widely employed due to their advantages over Euler angles, including avoidance of gimbal lock issues, higher computational efficiency, and reduced storage requirements [15,16,17]. The following outlines the derivation process for solving quaternions used in gyroscope-based attitude updates. First, the mathematical definition of quaternions is as follows:

(1) $$Q=q_{0}+q=q_{0}+q_{1}i+q_{2}j+q_{3}k=\cos\frac{\theta }{2}+u^{n}\sin\frac{\theta }{2}.$$

In the equation, $q_{0},q_{1},q_{2},q_{3}$ denote real numbers, while i, j, k represent the unit vector un indicating the rotation axis and direction, with θ denoting the rotation angle.

The angular velocity information output by the gyroscope is expressed as in Equation (2):

(2) $$\omega ={\left[\begin{array}{ccc} \omega_{x} & \omega_{y} & \omega_{z} \end{array}\right]}^{T}.$$

The relationship between quaternions and angular velocity may be expressed by the following equation:

(3) $$\left[\begin{array}{c} {\dot{q}}_{0}\\ {\dot{q}}_{1}\\ {\dot{q}}_{2}\\ {\dot{q}}_{3} \end{array}\right]=\frac{1}{2}\left[\begin{array}{cccc} 0 & -\omega_{x} & -\omega_{y} & -\omega_{z}\\ \omega_{x} & 0 & \omega_{z} & -\omega_{y}\\ \omega_{y} & -\omega_{z} & 0 & \omega_{x}\\ \omega_{z} & \omega_{y} & -\omega_{x} & 0 \end{array}\right]\left[\begin{array}{c} q_{0}\\ q_{1}\\ q_{2}\\ q_{3} \end{array}\right].$$

Solving Equation (3) yields the gyroscope-updated quaternion $q_{\omega }$ = ${\left[q_{0},q_{1},q_{2},q_{3}\right]}^{T}$. Substituting this into Equation (4) produces the attitude update matrix $C_{b}^{n}$:

(4) $$C_{b}^{n}=\left[\begin{array}{ccc} 1-2\left(q_{2}^{2}+q_{3}^{2}\right) & 2\left(q_{1}q_{2}-q_{0}q_{3}\right) & 2\left(q_{1}q_{3}+q_{0}q_{2}\right)\\ 2\left(q_{1}q_{2}+q_{0}q_{3}\right) & 1-2\left(q_{1}^{2}+q_{3}^{2}\right) & 2\left(q_{2}q_{3}-q_{0}q_{1}\right)\\ 2\left(q_{1}q_{3}-q_{0}q_{2}\right) & 2\left(q_{2}q_{3}+q_{0}q_{1}\right) & 1-2\left(q_{1}^{2}+q_{2}^{2}\right) \end{array}\right].$$

Equation (4) is denoted as Equation (5):

(5) $$C_{b}^{n}=\left[\begin{array}{ccc} T_{11} & T_{12} & T_{13}\\ T_{21} & T_{22} & T_{23}\\ T_{31} & T_{32} & T_{33} \end{array}\right].$$

Based on the conversion relationship between quaternions and euler angles, the attitude angles can be determined as shown in Equation (6):

(6) $$\left\{\begin{array}{c} \phi =\arctan\left(\frac{T_{12}}{T_{22}}\right)\\ \theta =\arcsin\left(T_{32}\right)\\ \gamma =\arctan\left(-\frac{T_{31}}{T_{33}}\right) \end{array}\right..$$

In the equation, ϕ, θ, and γ denote the heading angle, pitch angle, and roll angle respectively.

By mounting the measurement system described herein onto a moving carrier, with the positive direction of its X-axis accelerometer defined as the carrier’s forward direction, the displacement of the carrier along the X, Y, and Z axis directions can be determined by combining the carrier’s forward distance with its attitude angle information.

(7) $$\left\{\begin{array}{c} \Delta X=\cos(\Delta \theta )\cdot \cos(\Delta \phi )\cdot \Delta d\\ \Delta Y=\cos(\Delta \theta )\cdot \sin(\Delta \phi )\cdot \Delta d\\ \Delta Z=\sin(\Delta \theta )\cdot \Delta d \end{array}\right.$$

where Δd denotes the incremental forward distance, representing the difference between the current actual forward distance and the last stored actual forward distance; Δθ denotes the incremental pitch angle, representing the difference between the current actual pitch angle and the last stored actual pitch angle; Δϕ denotes the incremental heading angle, representing the difference between the current actual heading angle and the last stored actual heading angle; ΔX, ΔY, and ΔZ denote coordinate increments, representing the displacement changes in the three axes within the global coordinate system.

### 3.2. Zero-Bias Stability

Bias stability refers to the degree of random fluctuation in the output angular velocity or acceleration of an inertial sensor when stationary and free from interference. It constitutes a crucial performance metric for inertial sensors, reflecting their precision and reliability. Bias stability is typically expressed as the standard deviation of the output angular velocity over a specified time period. The output data of the inertial sensor is averaged using a smoothing time of 10 s. The specific calculation formula is as follows:

(8) $$B_{s}=\frac{1}{K}\sqrt{\frac{1}{N-1}\sum_{i=1}^{N}{\left(\omega_{i}-\omega \right)}^{2}},$$

(9) $$\omega =\frac{1}{N}\sum_{i=1}^{N}\omega_{i}.$$

Herein, $B_{s}$ represents the zero bias stability of the inertial sensor; *K* denotes the calibration factor of the inertial sensor; *N* signifies the number of data points smoothed over the specified time period; $\omega_{i}$ denotes the i-th data output from the inertial sensor following smoothing over the specified time period; ω and denotes the average value of the angular velocity or acceleration output by the inertial sensor.

### 3.3. Allan Variance

The Allan variance double-logarithmic curve plots the square root of Allan variance against the ratio of sampling time block length on a double-logarithmic coordinate system, revealing noise characteristics across different timescales. The specific calculation steps are as follows:

Step 1: Divide the entire data set into N blocks based on window length τ, with each block containing n data points and no overlap between blocks;

Step 2: Calculate the mean for the n data points within each block:

(10) $${\bar{y}}_{k}=\frac{1}{n}\sum_{i=k}^{k+n-1}y_{i}.$$

Step 3: Calculate the difference between adjacent blocks and compute the RMS value:

(11) $$\sigma^{2}\left(\tau \right)=\frac{1}{2(N_{c}-1)}\sum_{k=1}^{N_{c}-1}{\left({\bar{y}}_{k+1}-{\bar{y}}_{k}\right)}^{2}.$$

Step 4: Alter the value of n, repeat Steps 1–3, and plot a double-logarithmic curve showing the Allan standard deviation versus block length, which constitutes the Allan variance curve.

Based on the slope of the Allan variance curve, five typical error noise components can be identified as shown in Table 1: Quantisation Noise (QN), Angular Random Walk (ARW), Bias Instability (BI), Rate Random Walk (RRW), and Rate Ramp (RR). QN originates from errors during analogue-to-digital conversion, manifesting as subtle signal distortion. ARW primarily describes gyroscope angular drift caused by random noise over extended integration periods, constituting one of the principal error sources limiting sensor accuracy. BI typically presents as low-frequency noise. RRW denotes the random drift in the gyroscope’s angular velocity output caused by white noise; RR constitutes a deterministic error, exhibiting a time-dependent phenomenon primarily induced by the drift of physical parameters within the sensor’s internal structural components [18,19].

## 4. Experimental Validation and Analysis

**Figure 5:**
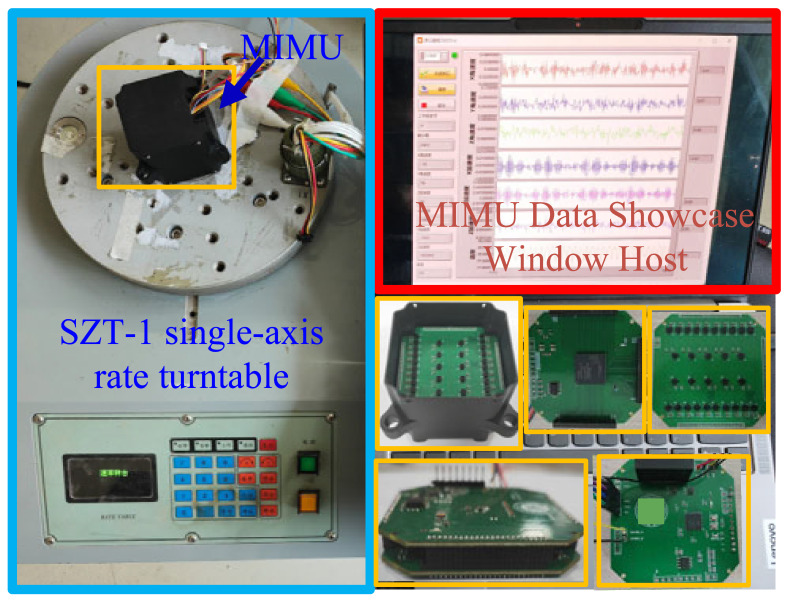
Test diagram of turntable for array MIMU acquisition system.

This experiment employs the SPI interface of an FPGA to communicate with the MIMU, transmitting data to an STM32F4 microcontroller. The STM32F4 parses, processes, and stores this data for subsequent retrieval. Upon completion of the test, commands are sent from the host computer to the microcontroller, which then transmits the stored data back via the RS422 bus. Data analysis is subsequently performed on the PC, forming a complete experimental system. The board-level circuit assembly platform for this experimental system is illustrated in Figure 5. Testing is conducted on a high-precision turntable, initially under static conditions followed by dynamic pipeline testing in realistic environments to validate the effectiveness of the measurement system. Throughout this process, the system operated at a supply voltage of 5 V, a current of 0.12 A, and a power consumption of 0.6 W.

### 4.1. Static Test Results

To evaluate the performance of the virtual gyroscope realised by the designed circuit, the MIMU data acquired from static experiments are first analysed. The experimental setup employed a sampling rate of 100 Hz, recording angular velocity data from both individual gyroscopes and the fusion of 60 gyroscopes. Angular velocity data spanning two hours of testing are extracted for processing.

**Figure 6:**
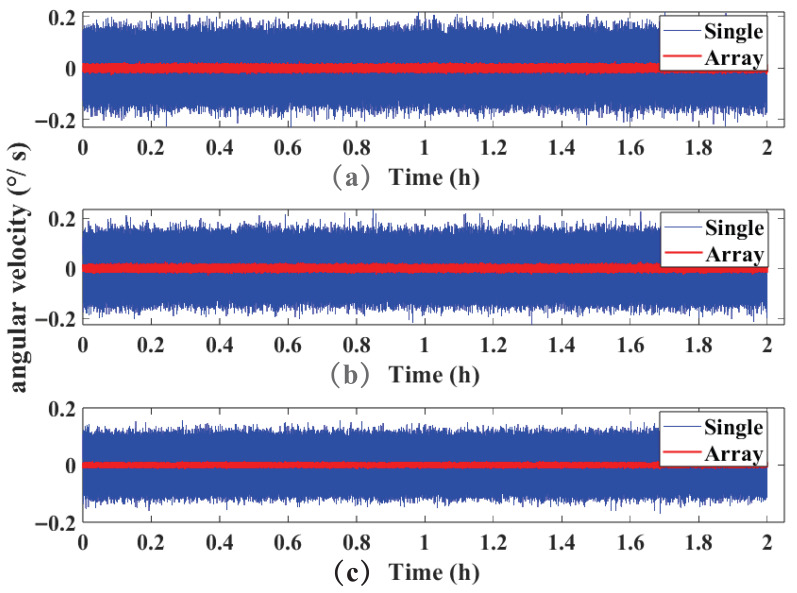
Comparison of angular velocity data for the X, Y, and Z axis: (**a**) angular velocity data for X-axis; (**b**) angular velocity data for Y-axis; (**c**) angular velocity data for Z-axis.

A visual comparison of the X, Y, and Z-axis angular velocity data from both individual gyroscopes and the fusion of 60 gyroscopes is shown in Figure 6. The calculated zero-bias stability for the X, Y, and Z axis of the single gyroscope are 6.795 °/h, 6.826 °/h, and 6.418 °/h respectively. For the 60-gyroscope fusion system, the corresponding values are 0.891 °/h, 0.893 °/h, and 0.867 °/h. The improvement factors for the X, Y, and Z axis array gyroscopes relative to a single gyroscope are 7.635, 7.644, and 7.403 respectively.

**Figure 7:**
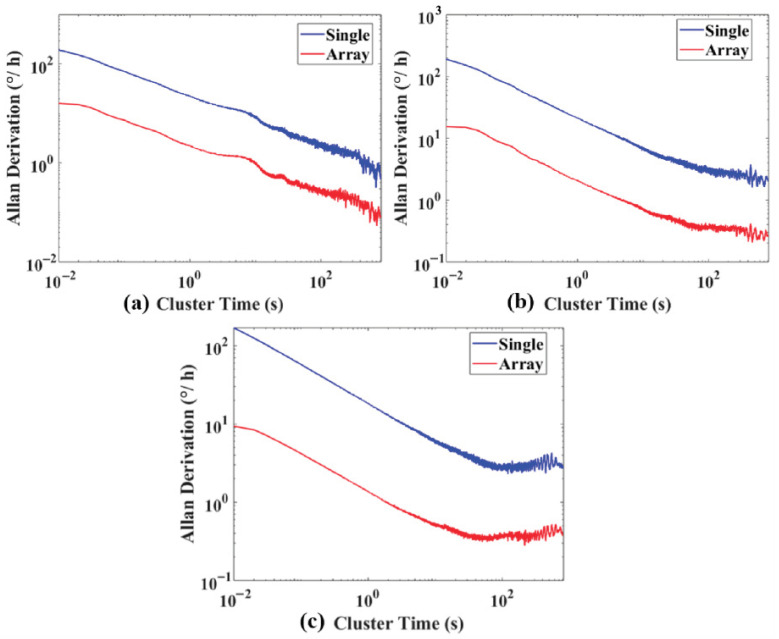
Allan variance plot comparisons: (**a**) X-axis; (**b**) Y-axis; (**c**) Z-axis.

By plotting the angular velocity double-logarithmic curve using Allan variance, as shown in Figure 7, quantitative analysis yielded specific values for ARW, BI, RRW, and others, as presented in Table 2. The comparison of typical random errors from Allan variance in Table 2 demonstrates that, compared to a single gyroscope, an array comprising 60 gyroscopes exhibits a significant advantage in suppressing random errors. The X-axis array gyroscope achieved a 7.67 times improvement over a single gyroscope in angular random walk; the Y-axis array gyroscope achieved a 7.587 times improvement; and the Z-axis array gyroscope achieved a 7.609 times improvement. Regarding zero-bias instability, the X, Y, and Z-axis array gyroscopes achieved improvement factors of 7.50, 7.439, and 7.583, respectively, compared to a single gyroscope. For rate random walk, the X, Y, and Z-axis array gyroscopes achieved improvement factors of 7.579, 7.572, and 7.390 respectively compared to a single gyroscope. Overall, the 60-gyroscope array demonstrated approximately 7.4–7.7 times improvements over individual gyroscopes for all three typical random error categories (ARW, BI, RRW) across the X, Y, and Z axis. This performance approaches the theoretical value of 7.74, conclusively validating the effectiveness of array design in mitigating gyroscope random errors.

### 4.2. Test Results for 100 m Pipeline

**Figure 8:**
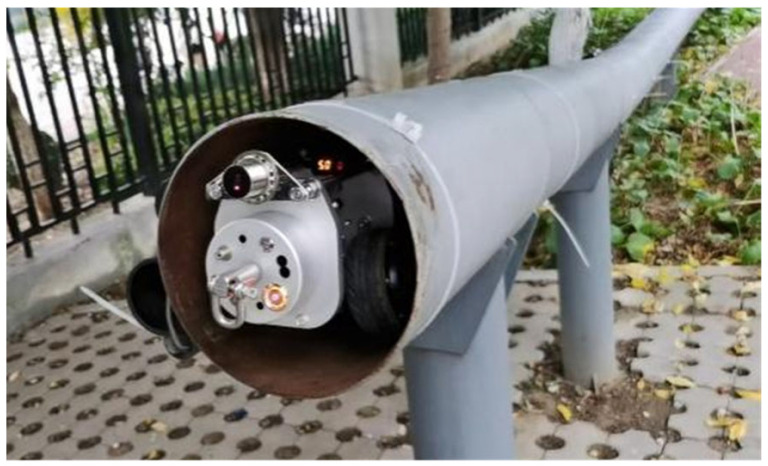
Standard pipeline actual test scenario.

To further demonstrate the practical performance of the array MIMU, measurements are conducted on a standard pipe, as shown in Figure 8. Due to the frequent presence of magnetic interference within pipelines, which inevitably affects magnetometers, selecting high-precision magnetometers and calibrating them through multi-position ellipsoidal fitting significantly reduces this impact. Additionally, during pipeline testing, we solely rely on magnetometers to determine the carrier’s initial heading angle. To further ensure data accuracy, multiple magnetic readings are collected during the magnetic data acquisition phase. The program automatically filters out outliers and averages the remaining magnetic data to guarantee the accuracy of the initial heading angle.

**Figure 9:**
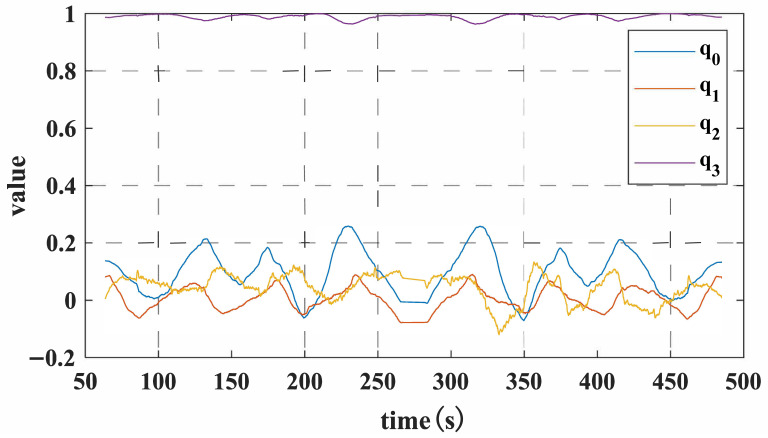
Round -trip measurement of quaternion data.

**Figure 10:**
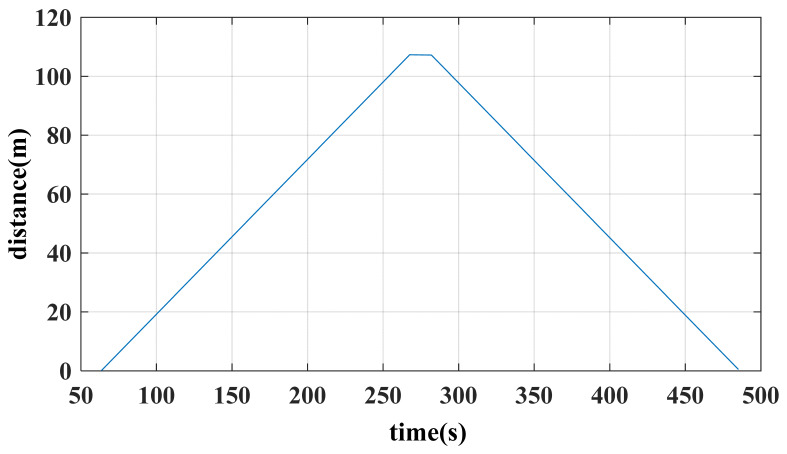
Round-trip measurement of positional data.

Mount the array MIMU on a servo-hub trolley to conduct bidirectional measurements along the pipeline, defining its X-axis as the trolley’s forward direction. Initially, determine the trolley’s initial attitude angles using the accelerometer and magnetometer. Subsequently, record angular velocity data and wheel travel distance every 10 cm of forward movement. Upon experiment completion, stored data is retrieved via the host computer to obtain quaternion and positional information, as illustrated in Figure 9 and Figure 10. Subsequent attitude calculation of the quaternion and positional data using MATLAB R2020a yields the pipeline’s actual measurement trajectory. However, this trajectory represents relative position coordinates; subsequent integration with the pipeline’s starting point coordinates is required to ultimately derive the pipeline’s absolute position coordinates.

**Figure 11:**
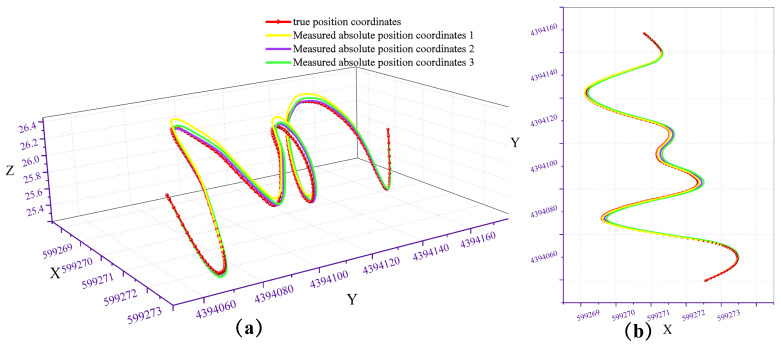
Comparison of the measured trajectory and the true trajectory: (**a**) 3D view; (**b**) top view.

**Figure 12:**
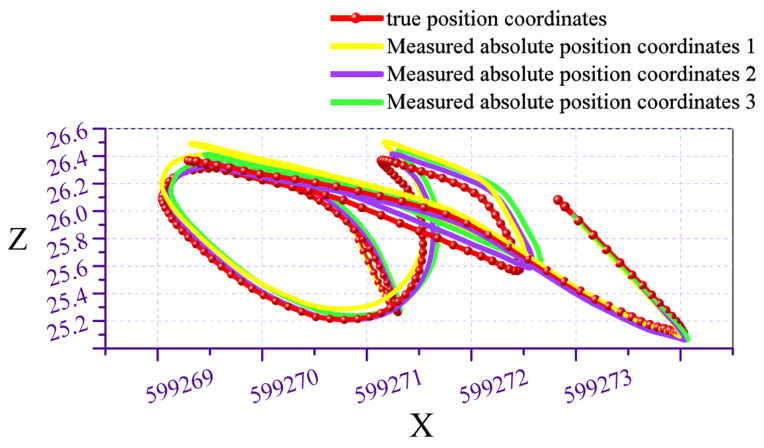
Comparison of measured trajectory with actual trajectory in front view.

**Figure 13:**
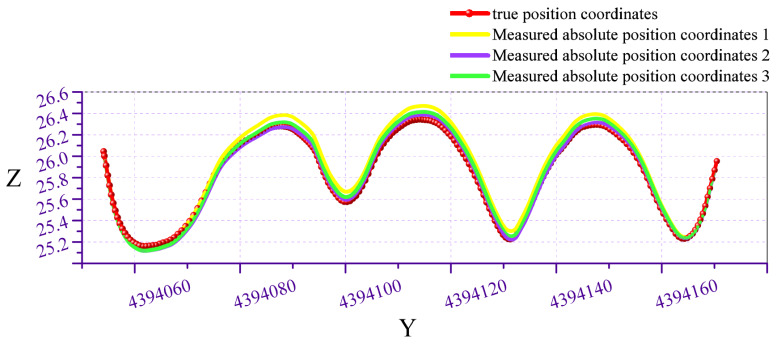
Side-view comparison of measured trajectory versus actual trajectory.

Repeating the experiment three times, the comparison between the measured absolute position coordinates and the true position coordinates is shown in Figure 11, Figure 12 and Figure 13. The red curve represents the pipeline’s true position coordinates, which were measured by the collaborating unit using millimeter-grade total stations and electronic levels in accordance with the Hebei Provincial Local Metrology Technical Specifications, while the other three coloured curves indicate the absolute position coordinates derived from processing the test data. Compared with the standard pipeline, the measured absolute position coordinates largely coincide with the true position coordinate curve. Calculations indicate a horizontal positioning error of less than 0.0774 m and an elevation positioning error of less than 0.0351 m.

## 5. Conclusions

This paper presents a real-time data acquisition system for array MIMU based on FPGA+ARM. It enables MIMU data reading, processing, multi-source information fusion, data storage, and communication with host computers, establishing a complete data link encompassing acquisition, transmission, processing, and storage. The paper details the overall system design (hardware circuit design and software code development), the construction of attitude update algorithms, and measurement accuracy evaluation metrics. Static experiments validated the system’s performance, demonstrating a 7.4 to 7.7 times improvement over a single gyroscope. In practical pipeline measurement applications, the system accurately calculated the position coordinates of a 100-m pipeline, achieving horizontal positioning errors below 0.0774 m and vertical positioning errors below 0.0351 m. This fully demonstrates the array design’s significant effectiveness in mitigating gyroscope random errors, providing a reliable technical solution for pipeline surveying and enabling its application in real-time scenarios demanding high precision. Subsequent work will prioritize the integration of real-time noise reduction algorithms based on Kalman filtering while strictly maintaining the system’s real-time performance. Specifically, this involves constructing a two-stage noise reduction architecture combining “array fusion + Kalman filtering”—using the hardware-level mean fusion from 60 MIMU channels as the observation input for Kalman filtering, and employing the filtering algorithm to dynamically estimate gyro random errors for real-time compensation. Next, optimize the adaptive filtering parameter adjustment strategy. Finally, embed this integrated algorithm into an FPGA to achieve a secondary enhancement in gyroscope measurement accuracy, further suppressing inherent gyroscope random errors. Simultaneously, expand its application scenarios to high-precision domains such as drones, robotics, and underground space exploration.

## Figures and Tables

**Table 1:** Five typical types of random error in Allan variance.

Noise Type	Symbol	Unit	Allan Standard Deviation
QN	Q	urad	$\sigma_{QN}\left(\tau \right)=\sqrt{3}Q/\tau$
ARW	N	${}^{\circ}/\sqrt{h}$	$\sigma_{ARW}\left(\tau \right)=N/\sqrt{\tau }$
BI	B	°/h	$\sigma_{B}\left(\tau \right)=\sqrt{2\ln2/\pi }B$
RRW	K	${}^{\circ}/h\sqrt{h}$	$\sigma_{RRW}\left(\tau \right)=\sqrt{\tau /3}K$
RR	R	${}^{\circ}/h^{2}$	$\sigma_{R}\left(\tau \right)=\tau R/\sqrt{2}$

**Table 2:** Allan variance comparisons of typical random errors.

Axis	Noise Type	Value	Improvement
X-axis	ARW (${}^{\circ}/\sqrt{h}$)	0.345 (Single), 0.045 (Array)	7.67 times
	BI (°/h)	2.214 (Single), 0.295 (Array)	7.50 times
	RRW (${}^{\circ}/h/\sqrt{h}$)	44.128 (Single), 5.822 (Array)	7.579 times
Y-axis	ARW (${}^{\circ}/\sqrt{h}$)	0.349 (Single), 0.046 (Array)	7.587 times
	BI (°/h)	2.321 (Single), 0.312 (Array)	7.439 times
	RRW (${}^{\circ}/h/\sqrt{h}$)	46.122 (Single), 6.091 (Array)	7.572 times
Z-axis	ARW (${}^{\circ}/\sqrt{h}$)	0.312 (Single), 0.041 (Array)	7.609 times
	BI (°/h)	1.873 (Single), 0.247 (Array)	7.583 times
	RRW (${}^{\circ}/h/\sqrt{h}$)	35.642 (Single), 4.823 (Array)	7.390 times

## Data Availability

The data used to support the findings of this study are available from the corresponding author upon request.
